# HiCPlotter integrates genomic data with interaction matrices

**DOI:** 10.1186/s13059-015-0767-1

**Published:** 2015-09-21

**Authors:** Kadir Caner Akdemir, Lynda Chin

**Affiliations:** Department of Genomic Medicine, Division of Cancer Medicine, The University of Texas MD Anderson Cancer Center, Houston, TX 77030 USA; Institute for Applied Cancer Science, The University of Texas MD Anderson Cancer Center, Houston, TX 77030 USA

## Abstract

Metazoan genomic material is folded into stable non-randomly arranged chromosomal structures that are tightly associated with transcriptional regulation and DNA replication. Various factors including regulators of pluripotency, long non-coding RNAs, or the presence of architectural proteins have been implicated in regulation and assembly of the chromatin architecture. Therefore, comprehensive visualization of this multi-faceted structure is important to unravel the connections between nuclear architecture and transcriptional regulation. Here, we present an easy-to-use open-source visualization tool, HiCPlotter, to facilitate juxtaposition of Hi-C matrices with diverse genomic assay outputs, as well as to compare interaction matrices between various conditions.

https://github.com/kcakdemir/HiCPlotter

**Electronic supplementary material:**

The online version of this article (doi:10.1186/s13059-015-0767-1) contains supplementary material, which is available to authorized users.

## Background

DNA is tightly packed inside the cell nucleus. Early light microscopy studies revealed that active chromatin forms different structures compared to heterochromatin [1]. Fine-scale identification of chromatin’s spatial organization has been empowered by chromatin conformation capture (3C)-based methods [2, 3]. The genome-wide chromosome conformation capture (Hi-C) assay elucidates chromosome folding on a genome-wide scale and generates interaction matrices that summarize contact probability between disparate stretches of chromatin [4]. Initial studies have highlighted the organization of the metazoan genome in three dimensions, where the somatic cell genome is compartmentalized into open (A) or closed (B) chromatin [5]. These compartments are tightly associated with transcriptional regulation and cell replication. Moreover, compartments are sub-structured into topologically associating domains (TADs) and chromatin loops [6–8]. These domains or loops strongly correlate with several “linear” genomic features, such as broad histone modifications (H3K9me2, H3K27me3), lamin A/B association, replication timing, DNase sensitivity or transcriptional activity [9, 10]. Various factors, including regulators of pluripotency binding such as Nanog and Klf4, long non-coding RNA (lincRNA) concentration, or the presence of “architectural proteins” (e.g., CTCF, Cohesin and Mediator), have been implicated in the regulation and assembly of chromatin architecture [11–15]. In addition, genomic structural alterations (e.g., copy number alterations and translocation events) can affect chromosomal domain integrity and therefore could alter proper regulation of transcription [16–20]. Therefore, visualization of various facets of chromatin regulation collectively will be important to augment our understanding of the complicated relationship between these different linear genomic features and chromatin’s spatial organization. A few Hi-C visualization tools exist [8, 21], but visualizing diverse genomic data types with interaction matrix data is still difficult, especially when accommodating different experimental conditions inside the same plot.

To meet these challenges, we developed an easy-to-use and open-source visualization tool, HiCPlotter, to facilitate the juxtaposition of Hi-C matrices with diverse genomic assay outputs, as well as to compare interaction matrices between various conditions. Importantly, we showcased HiCPlotter by applying it to publicly available interaction and genomic datasets, where we demonstrated how HiCPlotter can generate biological insights from readily available datasets. Here we show that cohesin long-range interactions coincide with the early replication DNA domains. Using HiCPlotter, we highlight a potentially important lincRNA locus that exhibits active chromatin formation in leukemia cell line K562 compared with normal blood cell line GM12878.

## Results and discussion

### Basic usage

**Fig. 1:**
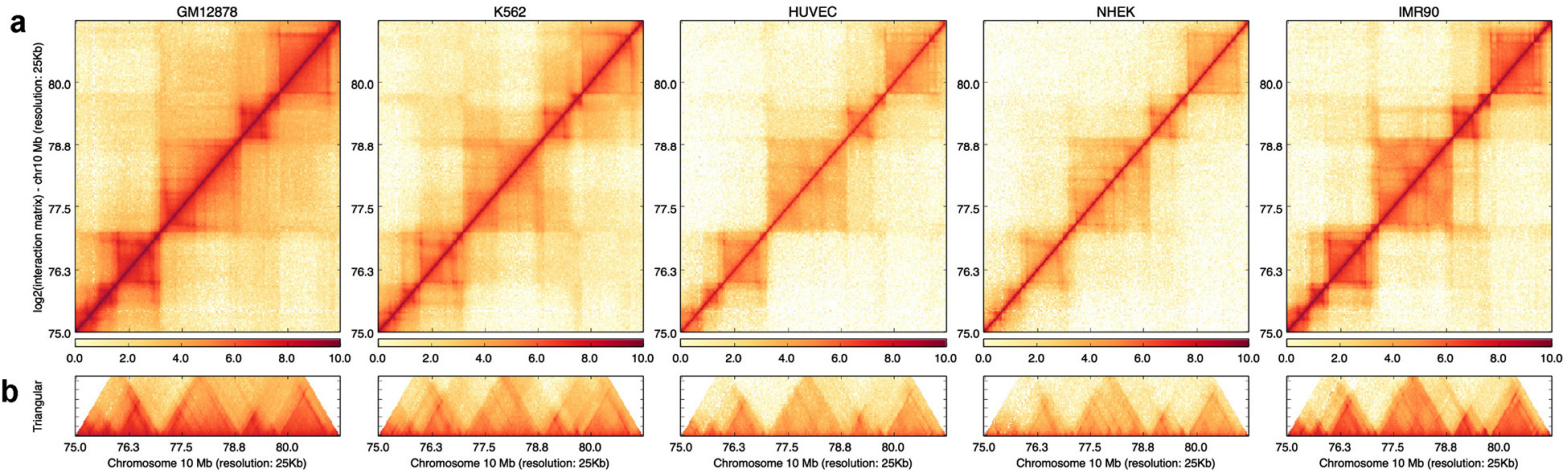
Basic usage of HiCPlotter. Genomic region inside human chromosome 10 as viewed with HiCPlotter. Interaction matrices of GM12878, K562, HUVEC, NHEK, and IMR90 cells can be displayed as a heatmap (**a**) and rotated half matrix (**b**), with the range of the rotated half matrix being 8 megabases from the diagonal

HiCPlotter requires an interaction matrix file, and is capable of displaying the data as an interaction matrix heatmap for a given chromosome (Additional file 1). Users can explore data with more detail by focusing on specific chromosomal subregions (Fig. 1). Several experimental conditions can be added and plotted next to others (Fig. 1a). Intrachromosomal interaction matrices are symmetrical; therefore, HiCPlotter can also represent the same data as a 45-degree rotated half matrix to facilitate better overlays with linear genomic features [22] (Fig. 1b). In addition, whole-genome interaction matrices or chromosome conformation capture carbon copy (5C) interaction matrices from different cell types can be plotted side-by-side (Additional files 2 and 3).

### Adding tracks

Tracks are individual plots that represent genomic features in genome browsers. Different aspects of the chromatin biology are captured by a wide spectrum of expanding biochemical assay outputs. Therefore, several tracks of a given experimental condition can be visualized for the same genomic coordinates (common x-axis) on top of each other for different genomic datasets. HiCPlotter is capable of plotting various assays’ outputs in different formats to enable capture of the best inherent genomic features.

**Fig. 2:**
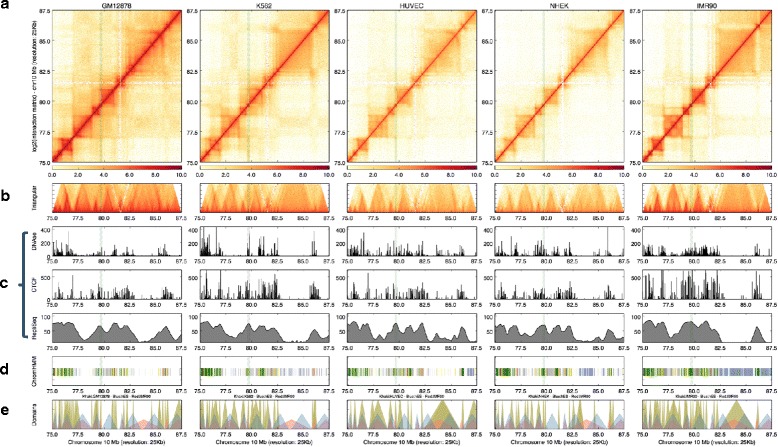
Adding tracks. Interaction matrices of GM12878, K562, HUVEC, NHEK, and IMR90 cells can be displayed as a heatmap (**a**) and rotated half matrix (**b**), with the range of the rotated half matrix being 8 megabases from the diagonal. **c** Histograms representing DNAseI hypersensitivity (*top*) and CTCF (*middle*) and Repli-Seq (*bottom*) signals for each type. **d** Tile plot of ChromHMM [20] calls within the represented locus. **e** Custom domain visualization as triangles. Arrowhead domains identified by Rao et al. [8] (*khaki*), and TADs reported [7] in human embryonic stem cells (*blue*) and IMR90 cells (*red*) are superimposed. An example arrowhead domain is highlighted by *green shaded column* inside the represented locus

Histograms are useful to visualize continuous data types along whole chromosomes, such as chromatin features or transcription factor binding (ChIP-Seq), open chromatin (DNase-Seq), replication-timing (Repli-Seq), lincRNA binding (RAP-Seq) and circular chromosome conformation capture (4C) assay outputs (Fig. 2c; Additional files 4 and 5). One key aspect of the histograms is that users can relate the coverage changes of a given assay with the higher-order chromatin context.

Tiles can be used to depict discrete genomic features, annotations, or analysis results, such as chromatin states [23] or domains, enhancer locations, and structural alterations (Fig. 2d; Additional file 6). Marking the locations of the specific annotations makes it easier to understand whether observed chromatin configurations overlap with the results of other genomic dataset analyses.

Arcs represent connectivity between two loci; this type of visualization is useful for assay outputs including 3C, ChIA-Pet to display enhancer–promoter pair loops [24], or “insulated neighborhoods” [19, 25] (Additional file 7). Chromatin domain borders are generally enriched with insulator proteins such as CP190 in *Drosophilia* or CTCF in mammals, and different architectural proteins are involved in chromatin looping at different length scales [26, 27]. Therefore, visualization of connectivity between genomic loci, especially in the higher-order chromatin context, is vital to understanding domain structures more clearly for certain cell types or under varying conditions.

Genomic material is folded into hierarchical layers and various types of folding structures have been proposed for the metazoan chromatin based on the length of the layers, such as chromosome compartments, TADs, or other chromosomal domains (e.g., arrowhead domains [8]). HiCPlotter enables display of custom domains as triangles that can be superimposed with previously published TADs for different human and mouse cells [7] (Fig. 2e).

In addition, emphasizing certain chromatin loci can be important, especially when the track number is high and focus is required. To accommodate this need, HiCPlotter allows users to highlight specific regions on the interaction matrix as well as additional tracks to augment the plot (Fig. 2). Looping between distant chromatin loci can also be annotated on the interaction matrix for pre-selected loci (Additional file 8).

### Late replicating genomic regions are depleted for cohesin connections

**Fig. 3:**
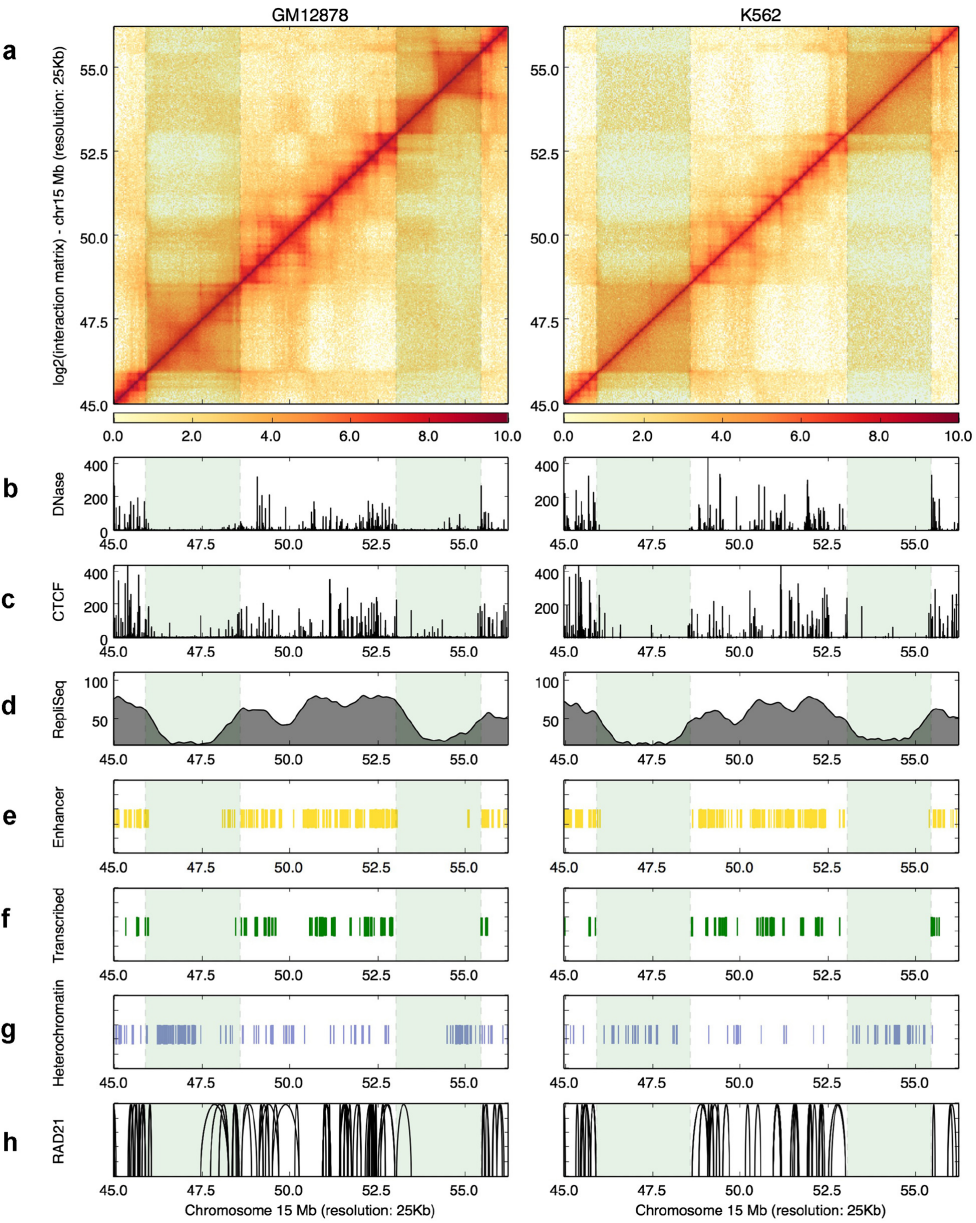
Cohesin ChIA-PET interactions coincide with early replication sites. **a** Hi-C contact maps are visualized as heatmaps for GM12878 and K562 cells. Histograms representing DNAseI hypersensitivity (**b**) and CTCF (**c**) and Repli-Seq (**d**) signals for each type. **e** Tiles mark enhancer calls with ChromHMM inside the visualized genomic segment. **f** Tiles mark transcribed regions identified with ChromHMM inside the visualized genomic segment. **g** Tiles mark heterochromatin regions identified with ChromHMM inside the visualized genomic segment. **h** Arcs visualize significantly interacting ChIA-PET tags for RAD21 inside the represented locus. Late replicating loci are highlighted by the two *green shaded columns*

To illustrate these visualization abilities of the HiCPlotter to reveal potentially interesting findings, we focused specifically on two hematopoietic cell lines profiled extensively by the ENCODE consortia, namely, an immortalized lymphoblastoid cell line (GM12878) and an immortalized chronic myelogenous leukemia cell line (K562). Data from ChIA-PET experiments targeting a subcomponent of the cohesin complex, RAD21, are also available for the aforementioned cell lines. Therefore, we deployed HiCPlotter’s arc plotting function to integrate long-range chromatin interaction data with other available data sets. ChIA-PET interactions are highly cell type-specific [28]; interestingly though, enriched RAD21-interacting regions were specifically observed at early replication domains but not in late replication DNA segments in both cell types (see highlighted region in Fig. 3). Almost half of the replication compartments are shared between cell types and late replicating compartments form larger chromosomal domains compared with the early replication compartments [29]. In addition, borders of topological domains overlap significantly with replication compartment borders, suggesting a connection between higher-order chromatin structure and DNA replication [30, 31]. Early replicating regions are generally transcriptionally and epigenetically more active compared with the late replicating DNA segments [29]. As shown in these two cells types, enhancer (Fig. 3e) or transcription loci (Fig. 3f) overlap with early replicating regions whereas heterochromatin loci coincide with late replication loci (Fig. 3g). To elucidate whether observed overlap between RAD21 ChIA-PET interactions and early replication compartments in GM12878 and K562 cells is specific just to the profiled region or is an inherent genomic feature of cohesin connectivity, we systematically analyzed all ChIA-PET interaction regions in terms of Repli-Seq signal. Compared with randomly selected loci or whole-genome distribution of Repli-Seq signal, RAD21-connected loci indeed overlap with higher Repli-Seq signal in both cell types (Figure S9a, b in Additional file 9). A similar trend is also observed between another core component of the cohesin complex, Smc1, ChIA-Pet interactions, and replication timing calculated thorough Repli-Chip assay in mouse embryonic stem cells (Figure S9c, d in Additional file 9), suggesting that the observed feature of cohesin interactions around the early replication domains is not restricted to a specific subunit of cohesin. In addition, this feature of cohesin is potentially conserved among mammals. This observation is in agreement with cohesin’s proposed role in DNA replication initiation by binding to DNA around replication origins and interacting with the pre-replication complex in order to stabilize loops around replication foci [32]. The binding of cohesin to chromatin throughout the cell cycle has been suggested to retain transcriptional memory by “bookmarking” the transcription factor binding sites [33]; in addition, defects in cohesin complex could lead to alterations of the cell cycle in the cell [34]. Our analysis revealed that connectivity between cohesin-to-cohesin long-range interaction sites occurs in early replicating regions of the chromatin, suggesting that, in addition to cohesin–DNA interactions, cohesin-to-cohesin interactions could play some architectural roles in interphase chromosomes and potentially influences both the transcription and cell cycle. This example demonstrates that visualization of various facets of chromatin with HiCPlotter could yield complementary insights to published findings from publicly available datasets.

### Potentially important lincRNAs for K562 cells

**Fig. 4:**
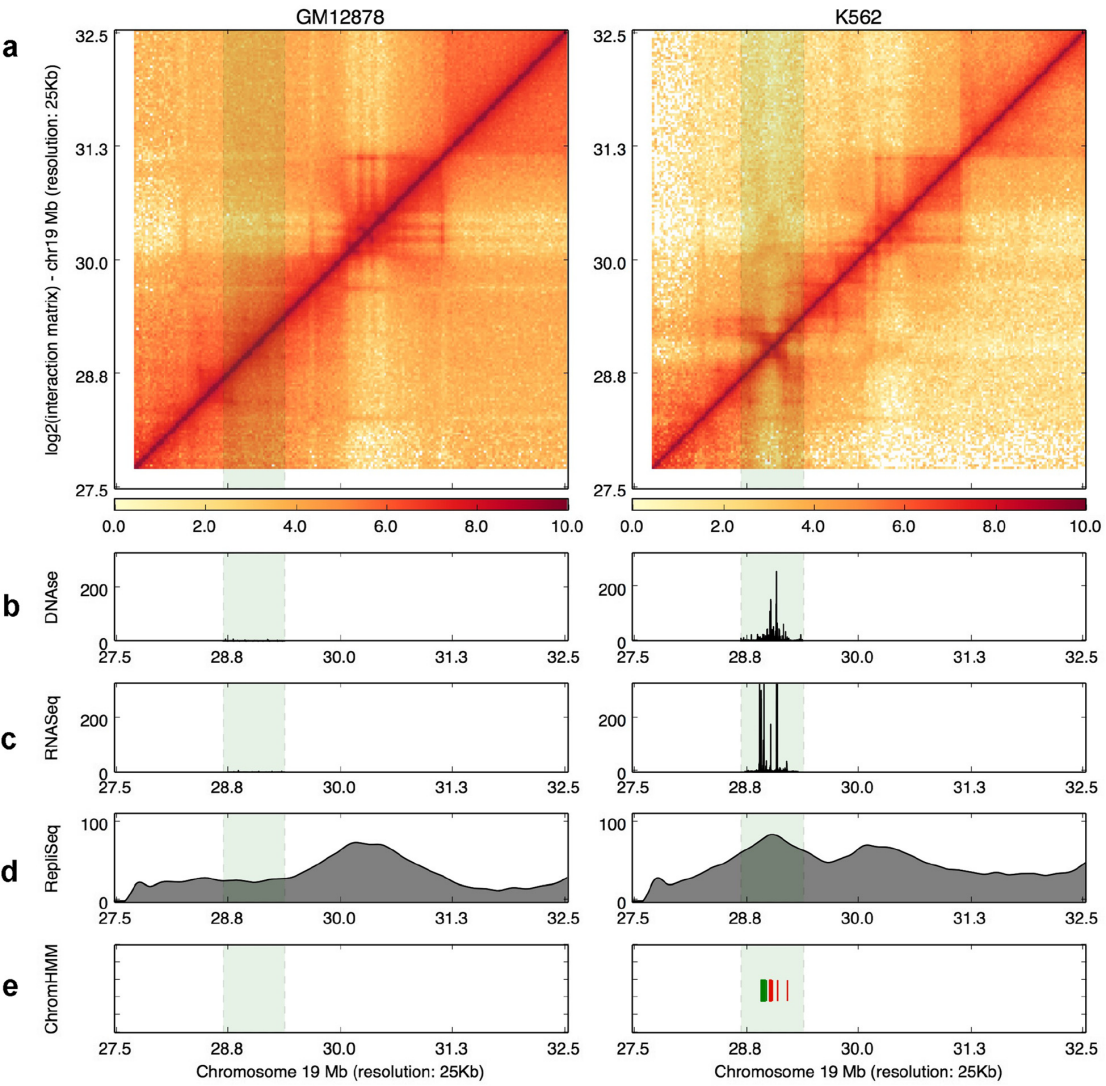
A lincRNA locus exhibits active chromatin formation in K562 cells. **a** Hi-C contact maps are visualized as heatmaps for GM12878 and K562 cells. Histograms representing DNAseI hypersensitivity (**b**), RNA-Seq expression (**c**), and Repli-Seq signals (**d**) for each type. **e** Tiles mark active transcription start site (*red*) and transcribed (*green*) state calls by ChromHMM inside the highlighted genomic segment. The lincRNA locus gaining active chromatin in K562 cells is highlighted by the *green shaded column*

Another feature of HiCPlotter is to enable side-by-side comparison of data from different experimental conditions, which helps to assess whether any specific change in observed genomic features is similarly reflected in higher-order chromatin interactions. To illustrate the usefulness of this, we sought to identify an example region that could be important for cell identity by comparing GM12878 and K562 cell line datasets. As both cell types originated from the same embryonic lineage and K562 is a malignant cell line, we looked for a region that exhibits different chromosomal and transcriptional outcome in K562 cells. A gene desert region on chromosome 19 hosts two lincRNAs. This region is silenced in GM12878 cells as no detectable RNA expression or DNaseI hypersensitivity is present (Fig. 4b, c). In addition, this locus seems to be part of a late replication domain in GM12878, as in Hi-C data a bigger domain is also observed. However, the same region exhibits strong RNA expression as well as DNase hypersensitivity in K562 cells (Fig. 4b, c). Replication timing seems to be shifted to early replication specifically around this region. More importantly, a specific TAD is formed surrounding this locus as observed in K562 Hi-C data (Fig. 4a, d). Chromatin states around this locus are also changed from heterochromatin in GM12878 cells to transcribed and active promoter states in K562 cells. In other words, our hypothesis that these lincRNAs are activated at this locus in K562 cells is supported by multiple assay outputs (Fig. 4e). However, changes in RNA expression, DNaseI hypersensitivity, or replication timing do not necessarily correlate with alterations in overall higher-order chromatin structure. As exemplified in another locus inside the same chromosome, RNA expression, DNaseI hypersensitivity, or replication timing do not correlate with any significant changes in Hi-C profiles of either GM12878 or K562 cells (Additional file 10). Similarly, specific changes in higher-order chromatin structure might not be reflected in other genomic assay outputs such as RNA-Seq or chromatin states (Additional file 11). These examples demonstrate that visualization of various facets of chromatin with HiCPlotter will help users sort through the significant changes observed under different conditions through the integration of various genomic features.

## Conclusions

Metazoan genome folding influences regulation of the DNA-based cellular functions. Therefore, understanding chromosome architecture in the context of various genomic features is critical. Comprehensive cataloging of genome folding is becoming practical with the advent of next-generation sequencing and the development of new conformation capture methods. NIH’s 4D Nucleome project aims to understand principles behind the nuclear architecture of chromatin by generating interaction maps from different cell types in both normal development and disease conditions. Therefore, there is a growing need for tools like HiCPlotter that adeptly accommodate various assay outputs within the same plot for different cell types or experimental conditions. We expect HiCPlotter will enable researchers to generate reproducible, coherent, intuitive, and high quality plots from increasingly available datasets. New features will be added continuously to HiCPlotter, based on user feedback and new genomic assay developments.

## Materials and methods

HiCPlotter is a command-line application written in Python with a minimum number of dependencies (namely numpy, matplotlib, and scipy) and generates coherent visual presentations of the data. It requires interaction matrix files, and is capable of displaying matrices as an interaction matrix (heatmap) and rotated half matrix (triangular plot). Additional tracks, imported from bedGraph format, can be displayed as histograms, tiles, arcs, or domains. HiCPlotter is released under MIT license.

### Data processing

Hi-C interaction datasets and arrowhead domain lists for GM12878, K562, HUVEC, NHEK, and IMR90 cell lines were downloaded from the Gene Expression Omnibus database (accession [GEO:GSE63525]). Matrices are generated for 25-kb resolution files by multiplying Knight and Ruiz normalization scores for two contacting loci and dividing raw observed values with this number as suggested by Rao et al. [8] for MAPQGE30 filtered reads. ENCODE project data for human (assembly hg19) and mouse (assembly mm9) cell lines were downloaded from the UCSC Genome Browser ENCODE portal. Bigwig signal files were converted to bedGraph format using Kent source utilities — bigWigToBegGraph executable [35]. Significantly interacting regions of RAD21 determined using ChIA-PET were obtained from Heidari et al. (supplementary table in [28]). Similarly, Smc1 ChIA-PET interacting loci were obtained from Dowen et al. (supplementary table S1E in [19]). Normalized mouse embryonic stem cell Hi-C matrices were downloaded from [7, 36]. TADs identified for human genome hg18 assembly [7] and recently lifted over to the hg19 assembly were downloaded from [37, 38]. Chromatin state calls (ChromHMM) were downloaded from [23, 39]. The remainder of the obtained datasets from published reports are listed in Additional file 12.

### Availability

The HiCPlotter source code and datasets used in this manuscript can be accessed at [40]. A sample run file (testRun.sh) is available and can be executed to generate outputs of HiCPlotter presented in this manuscript. Examples are selected to show how parameters of HiCPlotter can be tuned to exploit different genomic assay outputs and create coherent plots. For more information about each parameter, please visit the github page [40].

## Additional files

Additional file 1: Figure S1. Plotting a whole chromosome with HiCPlotter. Human chromosome 10 as viewed with HiCPlotter. Interaction matrices of GM12878, K562, HUVEC, NHEK, and IMR90 cells are displayed as heatmaps. (PDF 113 kb) Additional file 2: Figure S2. Whole genome plotting with HiCPlotter. Whole genome interaction profiles displayed with HiCPlotter in wild type (*WT*) T cells (*left*) and RAD21 knockout T cells (*right*). (PDF 187 kb) Additional file 3: Figure S3. Example visualization of 5C interaction data with HiCPlotter. Interaction matrices of mouse embryonic stem cells (*mESC*), mESCs with Xist deletion (*mESC_XO*) and mouse embryonic fibroblast cells (*MEF*) displayed as heatmaps (**a**) and rotated half matrix (**b**), in which the range of the rotated half matrix is 50 bins from the diagonal. (PDF 219 kb) Additional file 4: Figure S4. Example visualization of 4C and ChIP-Seq data as histograms with HiCPlotter. Interaction matrix of mouse embryonic stem cells displayed as heatmaps (**a**) and rotated half matrix (**b**), in which the range of the rotated half matrix is 8 megabases from the diagonal. **c** Histograms representing 4C genomic assay signals: four tracks are displayed for Hoxd4 interactions in mouse embryonic stem cells and mouse E9.5 tail bud, and Hoxd13 interactions in mouse embryonic stem cells and mouse E9.5 tail bud. **d** Histogram representing CTCF ChIP- Seq signal in mouse embryonic stem cells. **e** Custom domain visualization as triangles. TADs reported in mouse embryonic stem cells are displayed. (PDF 114 kb) Additional file 5: Figure S5. Example visualization of RAP-Seq data as histograms with HiCPlotter. **a** Interaction matrix of mouse embryonic stem cells displayed as a heatmap. **b** Histograms representing RAP-Seq genomic assay signals: five tracks are displayed for Xist localization across the X chromosome at five time points (*Xist_0h*, *Xist_1h*, *Xist_2h*, *Xist_3h*, and *Xist_6h*) after Xist induction. **c** Histogram representing H3K27me3 ChIP-Seq signal in mouse embryonic stem cells. (PDF 133 kb) Additional file 6: Figure S6. Example usage of tiles for presenting tissue-specific enhancer locations with HiCPlotter. **a** Interaction matrix of mouse embryonic stem cells displayed as a heatmap. **b** Histograms represent 4C genomic assay signals: two tracks are displayed for Hoxa13 interactions in mouse E12.5 digits (*Digits*) and in mouse E15.5 genitals (*GT*). **c** Tiles mark genital-specific (*red*), common (*blue*) and digit-specific (*green*) enhancer locations. **d** Custom domain visualization as triangles. TADs reported in mouse embryonic stem cells are displayed. (PDF 112 kb) Additional file 7: Figure S7. Example visualization of ChIA-Pet data as arcs with HiCPlotter. **a** Interaction matrix of mouse embryonic stem cells displayed as a heatmap. **b** Histograms represent ChIP-Seq assay signals: two tracks are displayed for CTCF and H3K27me3 in mouse embryonic stem cells. **c** Tile marking the polycomb domain in mouse embryonic stem cells. **d** Arcs represent high-confidence SMC1 ChIA-PET connected regions in mouse embryonic stem cells. (PDF 116 kb) Additional file 8: Figure S8. Annotating the interaction matrices by marking significantly interacting loci with *circles*. Interaction matrices of GM12878, KBM7, K562, HUVEC, IMR90, HMEC, and NHEK cells displayed as heatmaps. *Circles* (*cyan*) on the interaction matrices are identified as significantly interacting loci (HiCCUP peaks) and can be used for annotating certain regions on the matrices. (PDF 111 kb) Additional file 9: Figure S9. Cohesin ChIA-PET interactions are enriched in early replication domains. **a** Density plot represents distribution of Repli-Seq signal for RAD21 ChIA-PET ends (*blue*), whole genome distribution of Repli-Seq data (*red*), and randomly selected regions (*yellow*) in GM12878 cells. **b** Density plot representing distribution of Repli-Seq signal for RAD21 ChIA-PET ends (*blue*), whole genome distribution of Repli-Seq data (*red*), and randomly selected regions (*yellow*) in K562 cells. **c** Density plot represents distribution of Repli-Seq signal for Smc1 ChIA-PET ends (*blue*), whole genome distribution of Repli-Seq data (*red*), and randomly selected regions (*yellow*) in mouse embryonic stem cells. **d** An example region in mouse embryonic stem cells showing that Smc1 ChIA-PET interactions coincide with higher Repli-Chip signals. (PDF 130 kb) Additional file 10: Figure S10. An example region for no significant change in Hi-C maps even though linear genomic signal changes. **a** Hi-C contact maps are visualized as heatmaps for GM12878 and K562 cells. Histograms representing DNAseI hypersensitivity (**b**), RNA-Seq expression (**c**) and Repli-Seq signals (**d**) for each type. **e** Tiles mark chromatin states (*yellow* for enhancers, *green* for transcribed, and *purple* for heterochromatin regions) with ChromHMM inside the visualized genomic segment. The targeted locus is highlighted with the *green shaded column*. (PDF 157 kb) Additional file 11: Figure S11. An example region for no significant linear genomic signal changes even though genome architecture changes in the highlighted region. **a** Hi-C contact maps are visualized as heatmaps for GM12878 and K562 cells. Histograms representing DNAseI hypersensitivity (**b**), RNA-Seq expression (**c**) and Repli-Seq signals (**d**) for each type. **e** Tiles mark chromatin states (*yellow* for enhancers, *green* for transcribed, and *purple* for heterochromatin regions) with ChromHMM inside the visualized genomic segment. The targeted locus is highlighted with the *green shaded column*. (PDF 161 kb) Additional file 12: Table S1. Datasets used in this study. (PDF 115 kb)

## Abbreviations

3C: chromatin conformation capture
4C: circular chromosome conformation capture
5C: chromosome conformation capture carbon copy
lincRNA: long non-coding RNA
TAD: topologically associating domain
