# Supplementary figures and images for: HiCPlotter integrates genomic data with interaction matrices
Source: Genome Biol. 2015 Sep 21;16(1):198. doi: 10.1186/s13059-015-0767-1 (PMC4576377; doi:10.1186/s13059-015-0767-1)

Akdemir\_Additional file 1: Figure S1

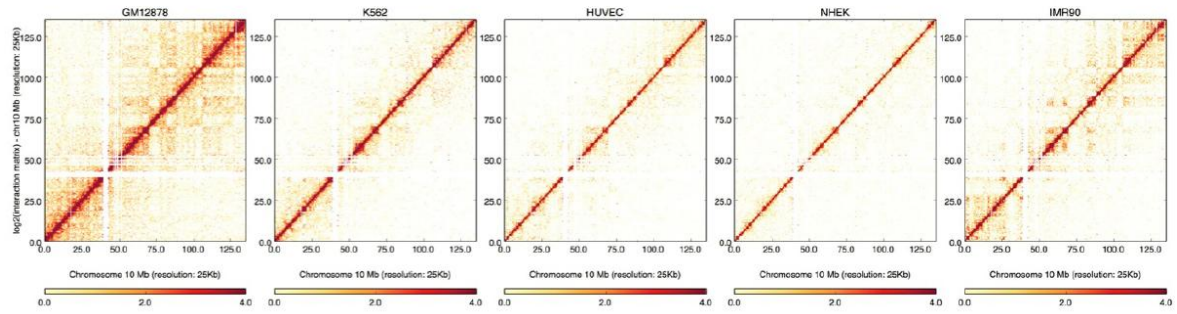

Supplement: Additional file 1: Figure S1. — Plotting a whole chromosome with HiCPlotter. Human chromosome 10 as viewed with HiCPlotter. Interaction matrices of GM12878, K562, HUVEC, NHEK, and IMR90 cells are displayed as heatmaps. (PDF 113 kb) [file 13059_2015_767_MOESM1_ESM.pdf]

Akdemir\_Additional file 2: Figure S2

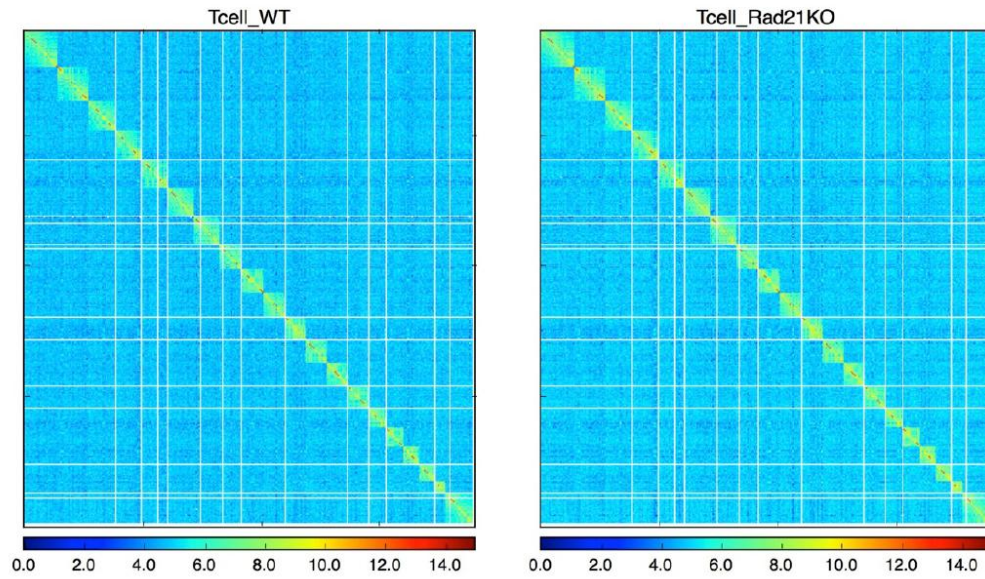

Supplement: Additional file 2: Figure S2. — Whole genome plotting with HiCPlotter. Whole genome interaction profiles displayed with HiCPlotter in wild type (WT) T cells (left) and RAD21 knockout T cells (right). (PDF 187 kb) [file 13059_2015_767_MOESM2_ESM.pdf]

Akdemir\_Additional file 3: Figure S3

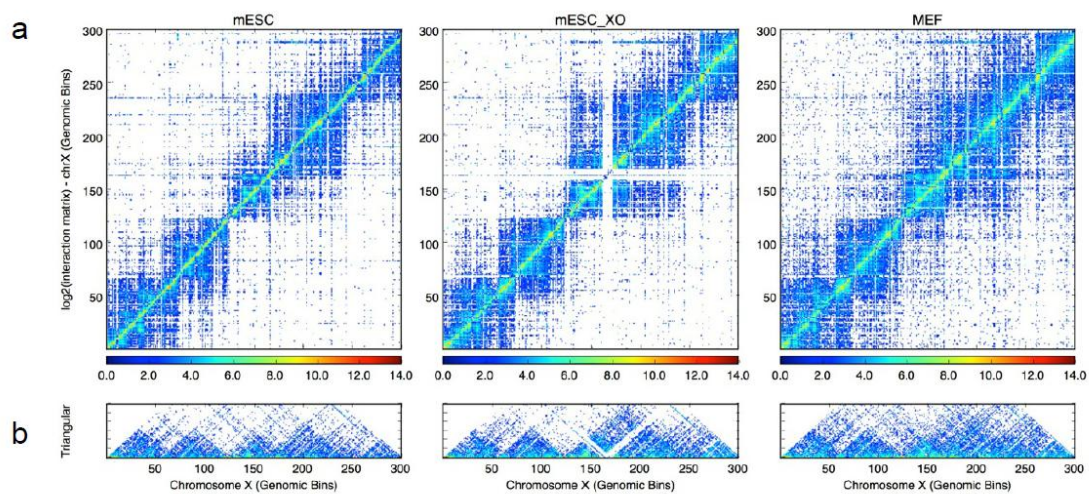

Supplement: Additional file 3: Figure S3. — Example visualization of 5C interaction data with HiCPlotter. Interaction matrices of mouse embryonic stem cells (mESC), mESCs with Xist deletion (mESC_XO) and mouse embryonic fibroblast cells (MEF) displayed as heatmaps (a) and rotated half matrix (b), in which the range of the rotated half matrix is 50 bins from the diagonal. (PDF 219 kb) [file 13059_2015_767_MOESM3_ESM.pdf]

Akdemir\_Additional file 4: Figure S4

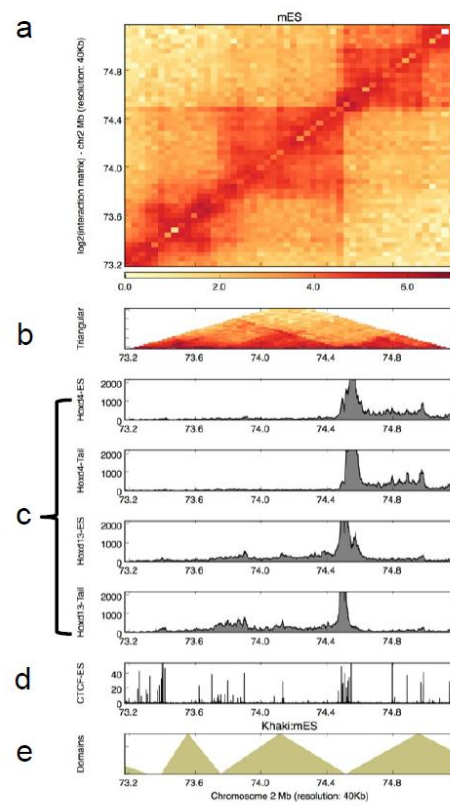

Supplement: Additional file 4: Figure S4. — Example visualization of 4C and ChIP-Seq data as histograms with HiCPlotter. Interaction matrix of mouse embryonic stem cells displayed as heatmaps (a) and rotated half matrix (b), in which the range of the rotated half matrix is 8 megabases from the diagonal. c Histograms representing 4C genomic assay signals: four tracks are displayed for Hoxd4 interactions in mouse embryonic stem cells and mouse E9.5 tail bud, and Hoxd13 interactions in mouse embryonic stem cells and mouse E9.5 tail bud. d Histogram representing CTCF ChIP- Seq signal in mouse embryonic stem cells. e Custom domain visualization as triangles. TADs reported in mouse embryonic stem cells are displayed. (PDF 114 kb) [file 13059_2015_767_MOESM4_ESM.pdf]

Akdemir\_Additional file 5: Figure S5

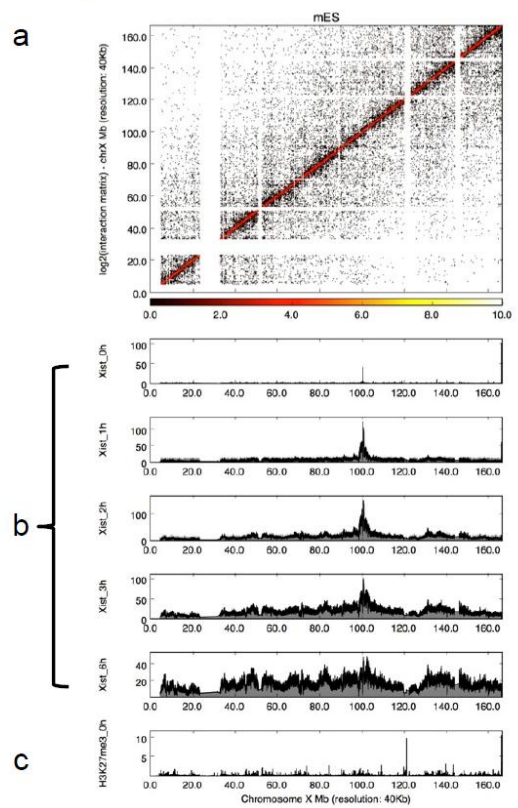

Supplement: Additional file 5: Figure S5. — Example visualization of RAP-Seq data as histograms with HiCPlotter. a Interaction matrix of mouse embryonic stem cells displayed as a heatmap. b Histograms representing RAP-Seq genomic assay signals: five tracks are displayed for Xist localization across the X chromosome at five time points (Xist_0h, Xist_1h, Xist_2h, Xist_3h, and Xist_6h) after Xist induction. c Histogram representing H3K27me3 ChIP-Seq signal in mouse embryonic stem cells. (PDF 133 kb) [file 13059_2015_767_MOESM5_ESM.pdf]

Akdemir\_Additional file 6: Figure S6

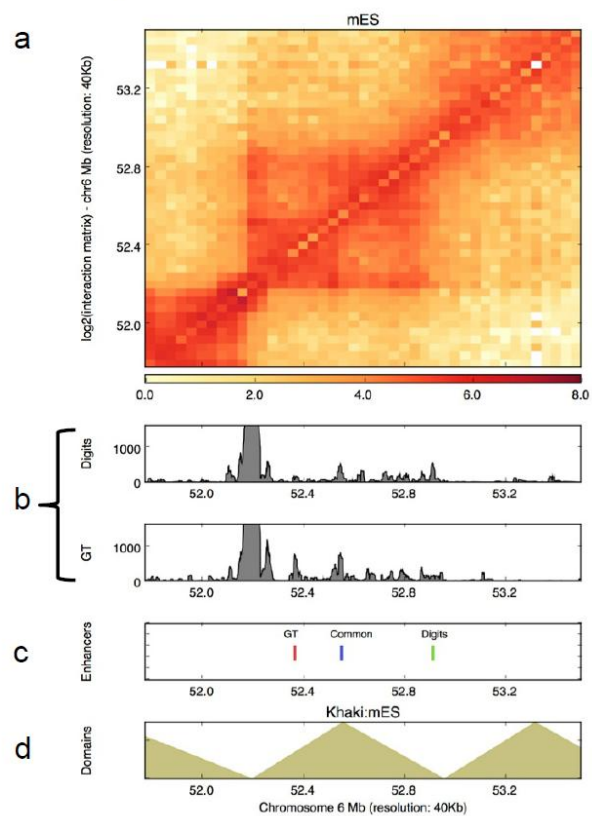

Supplement: Additional file 6: Figure S6. — Example usage of tiles for presenting tissue-specific enhancer locations with HiCPlotter. a Interaction matrix of mouse embryonic stem cells displayed as a heatmap. b Histograms represent 4C genomic assay signals: two tracks are displayed for Hoxa13 interactions in mouse E12.5 digits (Digits) and in mouse E15.5 genitals (GT). c Tiles mark genital-specific (red), common (blue) and digit-specific (green) enhancer locations. d Custom domain visualization as triangles. TADs reported in mouse embryonic stem cells are displayed. (PDF 112 kb) [file 13059_2015_767_MOESM6_ESM.pdf]

Akdemir\_Additional file 7: Figure S7

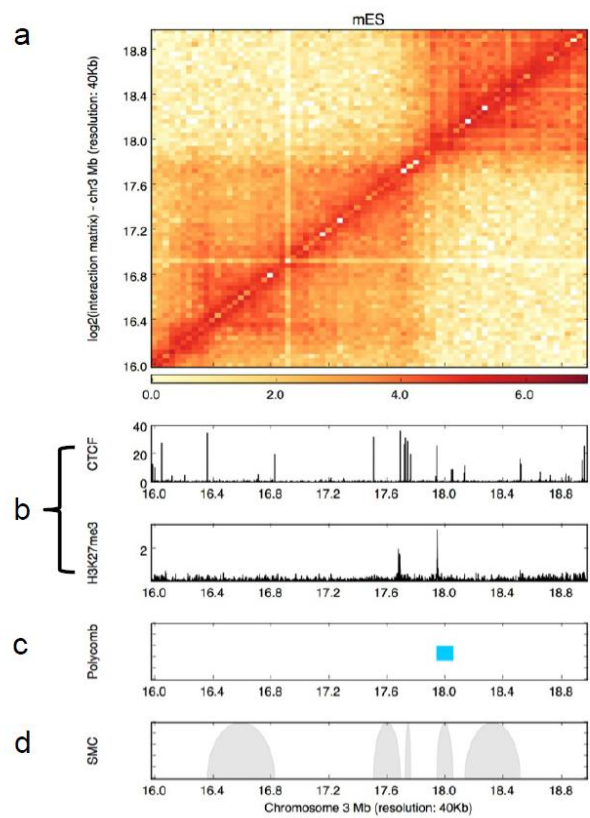

Supplement: Additional file 7: Figure S7. — Example visualization of ChIA-Pet data as arcs with HiCPlotter. a Interaction matrix of mouse embryonic stem cells displayed as a heatmap. b Histograms represent ChIP-Seq assay signals: two tracks are displayed for CTCF and H3K27me3 in mouse embryonic stem cells. c Tile marking the polycomb domain in mouse embryonic stem cells. d Arcs represent high-confidence SMC1 ChIA-PET connected regions in mouse embryonic stem cells. (PDF 116 kb) [file 13059_2015_767_MOESM7_ESM.pdf]

Akdemir\_Additional file 8: Figure S8

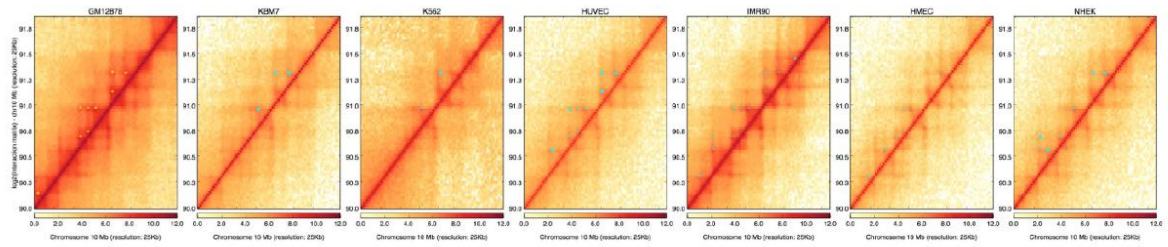

Supplement: Additional file 8: Figure S8. — Annotating the interaction matrices by marking significantly interacting loci with circles. Interaction matrices of GM12878, KBM7, K562, HUVEC, IMR90, HMEC, and NHEK cells displayed as heatmaps. Circles (cyan) on the interaction matrices are identified as significantly interacting loci (HiCCUP peaks) and can be used for annotating certain regions on the matrices. (PDF 111 kb) [file 13059_2015_767_MOESM8_ESM.pdf]

## Akdemir\_Additional file 9: Figure S9

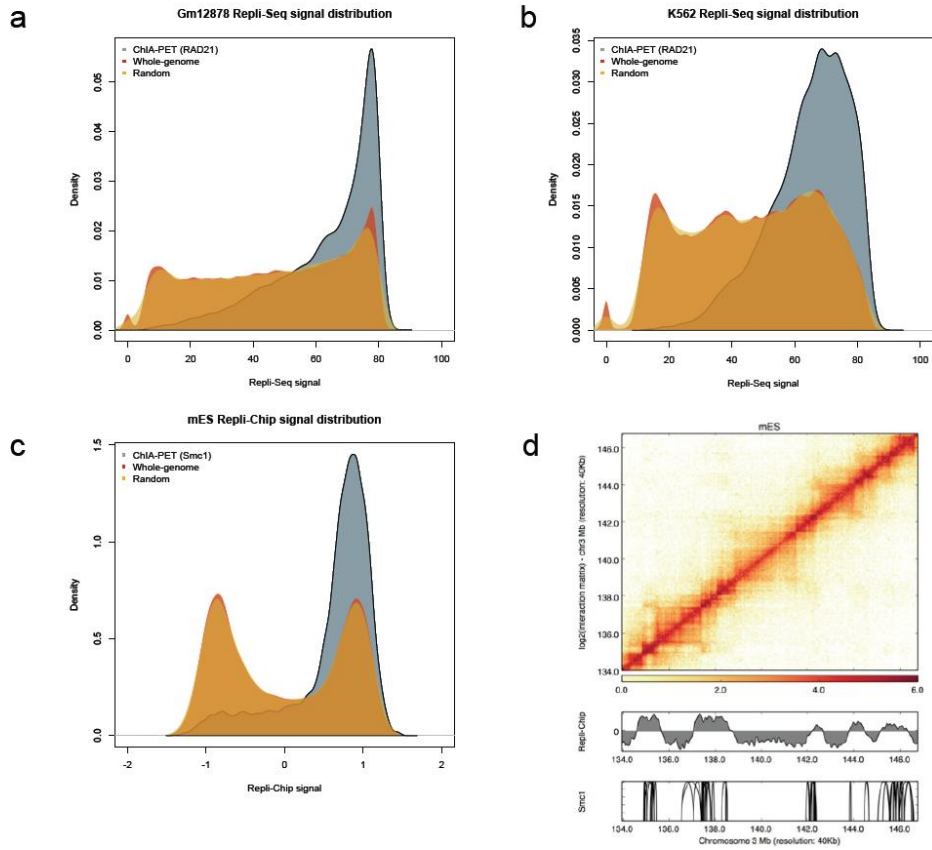

Supplement: Additional file 9: Figure S9. — Cohesin ChIA-PET interactions are enriched in early replication domains. a Density plot represents distribution of Repli-Seq signal for RAD21 ChIA-PET ends (blue), whole genome distribution of Repli-Seq data (red), and randomly selected regions (yellow) in GM12878 cells. b Density plot representing distribution of Repli-Seq signal for RAD21 ChIA-PET ends (blue), whole genome distribution of Repli-Seq data (red), and randomly selected regions (yellow) in K562 cells. c Density plot represents distribution of Repli-Seq signal for Smc1 ChIA-PET ends (blue), whole genome distribution of Repli-Seq data (red), and randomly selected regions (yellow) in mouse embryonic stem cells. d An example region in mouse embryonic stem cells showing that Smc1 ChIA-PET interactions coincide with higher Repli-Chip signals. (PDF 130 kb) [file 13059_2015_767_MOESM9_ESM.pdf]

Akdemir\_Additional file 10: Figure S10

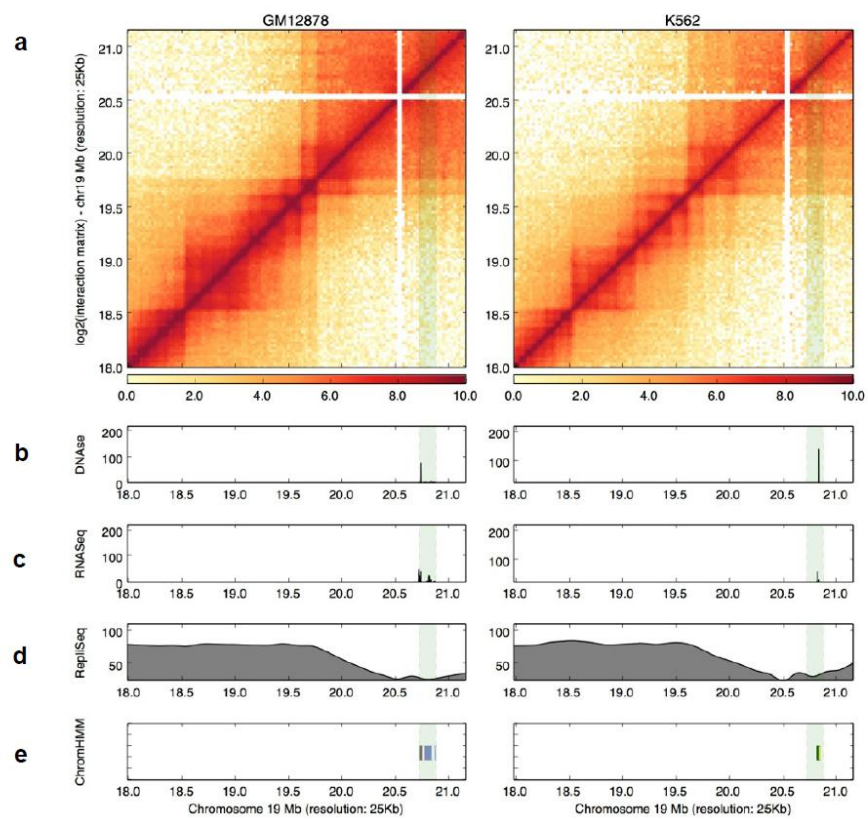

Supplement: Additional file 10: Figure S10. — An example region for no significant change in Hi-C maps even though linear genomic signal changes. a Hi-C contact maps are visualized as heatmaps for GM12878 and K562 cells. Histograms representing DNAseI hypersensitivity (b), RNA-Seq expression (c) and Repli-Seq signals (d) for each type. e Tiles mark chromatin states (yellow for enhancers, green for transcribed, and purple for heterochromatin regions) with ChromHMM inside the visualized genomic segment. The targeted locus is highlighted with the green shaded column. (PDF 157 kb) [file 13059_2015_767_MOESM10_ESM.pdf]

Akdemir\_Additional file 11: Figure S11

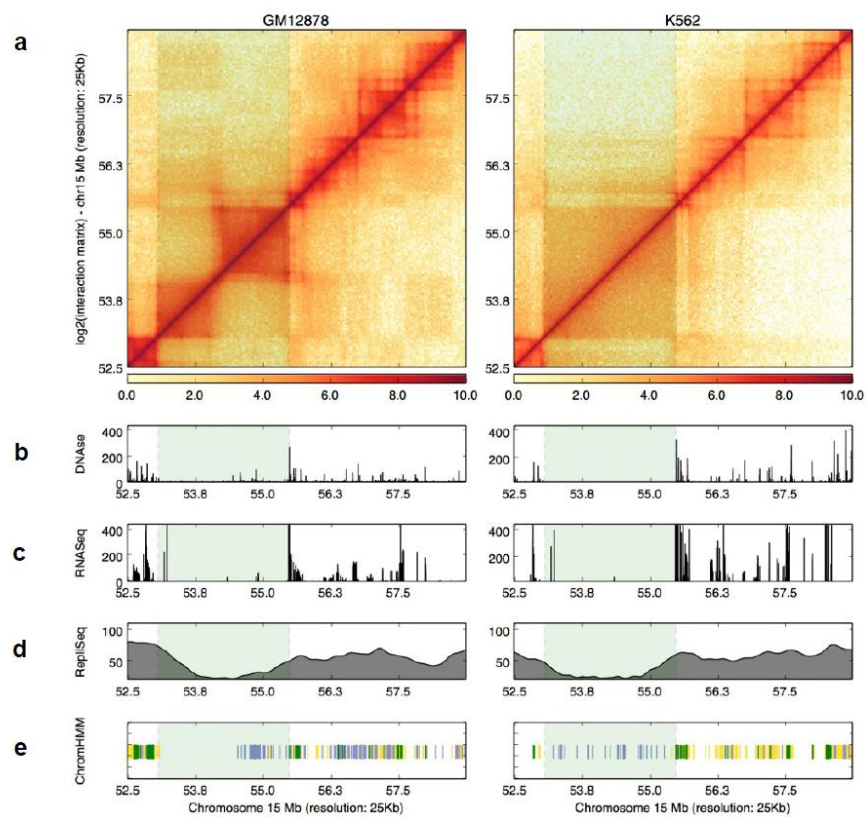

Supplement: Additional file 11: Figure S11. — An example region for no significant linear genomic signal changes even though genome architecture changes in the highlighted region. a Hi-C contact maps are visualized as heatmaps for GM12878 and K562 cells. Histograms representing DNAseI hypersensitivity (b), RNA-Seq expression (c) and Repli-Seq signals (d) for each type. e Tiles mark chromatin states (yellow for enhancers, green for transcribed, and purple for heterochromatin regions) with ChromHMM inside the visualized genomic segment. The targeted locus is highlighted with the green shaded column. (PDF 161 kb) [file 13059_2015_767_MOESM11_ESM.pdf]
