# Supplementary material for: HiCPlotter integrates genomic data with interaction matrices
Source: Genome Biol. 2015 Sep 21;16(1):198. doi: 10.1186/s13059-015-0767-1 (PMC4576377; doi:10.1186/s13059-015-0767-1)
Supplement: Additional file 12: Table S1. — Datasets used in this study. (PDF 115 kb) [file 13059_2015_767_MOESM12_ESM.pdf]

Akdemir_Additional file 12: Table S1 : Datasets Used in This Study

Publication	Year	Journal	PMID	Accession
Topological Domains In Mammalian Genomes Identified By Analysis Of Chromatin Interactions	2012	Nature	22495300	GSE35156
Cohesin And Ctcf Differentially Affect Chromatin Architecture And Gene Expression In Human Cells	2014	PNAS	24335803	GSE44267
Temporal Dynamics And Developmental Memory Of 3d Chromatin Architecture At Hox Gene Loci	2014	eLife	24843030	GSE55344
Dna-Binding Factors Shape The Mouse Methylome At Distal Regulatory Regions	2011	Nature	22170606	GSE30206
A Comparative Encyclopedia Of Dna Elements In The Mouse Genome	2014	Nature	25409824	GSE49847
The Xist Lncrna Exploits Three-Dimensional Genome Architecture To Spread Across The X Chromosome	2013	Science	23828888	GSE46918
Mapping And Analysis Of Chromatin State Dynamics In Nine Human Cell Types	2011	Nature	21441907	GSE26386
Control Of Cell Identity Genes Occurs In Insulated Neighborhoods In Mammalian Chromosomes	2014	Cell	25303531	GSE57913
Convergent Evolution Of Complex Regulatory Landscapes And Pleiotropy At Hox Loci	2014	Science	25414315	GSE62269
A 3d Map Of The Human Genome At Kilobase Resolution Reveals Principles Of Chromatin Looping	2014	Cell	25497547	GSE63525
Genome-Wide Map Of Regulatory Interactions In The Human Genome	2014	Genome Research	25228660	GSE59395
Cohesin-Based Chromatin Interactions Enable Regulated Gene Expression Within Preexisting Architectural Compartments	2014	Genome Research	24002784	GSE48763
Spatial Partitioning Of The Regulatory Landscape Of The X-Inactivation Centre	2014	Nature	22495304	GSE35721
Integrative analysis of 111 reference human epigenomes	2015	Nature	25693563	http://compbio.mit.edu/roadmap/
Topologically Associating Domains Are Stable Units Of Replication-Timing Regulation	2014	Nature	25409831	GSE51334
